# Vacation for Doctors

**Published:** 1886-08

**Authors:** 


### VACATION FOR DOCTORS.

If there be any truth in the old adage, "*laborare est orare*," it is certain that medical men constitute the most devout class of people on the face of the earth. It is a fact almost axiomatic in its truth that the physician in full general practice works harder for his living than any other laborer, either professional or non-professional. It is easily demonstrable that in the long run brain-work is more exhausting and devitalizing in its effects than any kind of manual labor. Statistics show that the expectation of life is less in physicians than in any other of the professional classes. In other words, it is plain that the majority of doctors work themselves to death. The demands of a large *clientele*, the reading and study which are necessary to keep abreast with the progress of medicine and the journalistic and society work which a sense of duty should impose upon every member of the profession, make up a sum total whose magnitude would be appalling were it not looked upon as inevitable and taken as a matter of course.

As long as a physician remains at home it is impossible for him to escape from his work. It will follow him and seek him out wherever he goes. There is but one resource for him—he must drop everything and fly. If he waits for his work to assume such shape that he can leave it, he will wait until death gives him a longer vacation than he asks for. It is true that a

Hammond and a Sir Henry Thompson may find leisure for writing novels, but these men do not represent the rank and file of the profession. The busy practitioner of medicine is a modern Sisyphus, who, though he tug and toil forever, will never succeed in rolling the stone to the top of the hill.

A muscle may, by continuous exercise, be developed up to a certain point; but if the training process be carried beyond that point degeneration occurs. This fact is well known to athletes, who always guard against over-training. The same rule applies to the higher nerve-centres, whose functional activity is correlative with mental labor. Within certain limits their exercise is conducive to vigor and health. Beyond those limits it leads to degeneration and decay. This degeneration may show itself in the nerve-centres themselves, or it may appear as irremediable perversions of nutrition in other parts of the body, constituting organic disease. By virtue of this law physicians wear out and die at an age when other men are in their prime.

What is the remedy for this state of things? It evidently lies in relaxing the bow occasionally, instead of keeping it always bent. A trip to the mountains or the sea-shore, a run over to Europe, anything which will break up the monotony and weariness of the daily routine of practice, will furnish new pabulum for the worn-out nerve-centres to replace that which has been exhausted by the tread-mill round of professional toil. A moderate amount of judicious dissipation may be wisely indulged in and will be productive of good, physically, mentally, and perhaps morally. The brain will thus regain its clearness and force, the body will be re-invigorated, the blood will course through the arteries with new energy, the hand will be firmer and steadier for its work, the whole man will be rejuvenated and renewed, so that if the experiment be made an annual one, a score of years of health and usefulness may be added to one's life. Such results are certainly

*desiderata* to all who value comfort and happiness, and they can be obtained only in the way that we have indicated. We commend the subject to the consideration of every medical man, whether he rolls in his coupé through the fashionable avenues of the city, or jogs along with his saddle-bags over the dusty roads of the rural districts, for to the one as to the other it is in the long run a matter of vital importance.

---

### ITEMS.

DR. J. C. AVARY, of this city, has been quite sick, but is out again.

THE City Council has employed a physician to attend the sick prisoners at the police station for $10.00 a month. How extravagant!

WE pity the doctor who can't do the smallest surgical operation or visit a patient of any prominence without running to the newspapers with it.

MALTINE is now improved by being of a more fluid consistency and agreeable to the taste; it is also combined with cascara sagrada as a mild laxative.

DR. HUNTER P. COOPER, of Atlanta, Ga., lately of this city, has been made an adjunct professor of chemistry in the Atlanta Medical College. We congratulate the doctor and the college. —*N. Y. Medical Record.*

THE *Nouvelles Archives d'Obstetrique et de Gynecologie*, of June 25, publishes an abstract of Dr. Virgil O. Hardon's paper on "The Use and Abuse of Pessaries," and credits it to the *Medical News.* We take the liberty of calling the attention of Dr. Zinowieff to the fact that the article appeared in the ATLANTA MEDICAL AND SURGICAL JOURNAL, of December, 1885.

THE *St. Louis Medical and Surgical Journal*, one of our best exchanges, entered upon its fifty-first year in July. It is the oldest medical monthly in the United States.

WE had a pleasant call a few days ago from Mr. J. W. Angier, representing the well-known publisher and book-seller, E. R. Pelton, New York. Mr. Pelton is the publisher of the *Eclectic Magazine*, one of the best literary publications in America.

SEVERAL Atlanta physicians are off on a vacation. Dr. J. S. Todd and family are at Cumberland Island; Dr. W. S. Armstrong and family are at Porter Springs; Dr. W. S. Elkin is at Crab Orchard Springs, Ky., and Dr. H. V. M. Miller and wife are at Heywood White Sulphur Springs in Western North Carolina.

A MISTAKE CORRECTED.—Dr. Hunter P. Cooper, Atlanta, writes as follows, correcting an error which appeared in our last issue: "In the July number of your excellent JOURNAL, you mention my appointment as Adjunct Professor of Chemistry in the Atlanta Medical College, but you make a mistake in stating that I was formerly connected with the 'Presbyterian Eye and Ear Hospital of New York.' The Presbyterian Hospital in which I served is a *General Medical and Surgical* Hospital, and not devoted to any specialty. Please make this correction and oblige."

DR. F. NORTON MANNING (*Medical Abstract*), in a paper on the study of heredity, says: In connection with these cases, I may mention that Dr. Bemiss obtained particulars of 31 children born in the United States, of brother and sister, or parent and child, and of these 29 were defective in one way or another, 19 were idiotic, 1 epileptic, 5 scrofulous, and 11 deformed; and the same inquirer found that of 2,778 children born of first cousins, 793 were defective, 117 deaf and dumb, 63 blind, 231 idiotic, 24 insane, 44 epileptic, 189 scrofulous, and 9 deformed.

ATLANTA has the best newspaper reporters in the world. They never fail to find out when a person from a distance comes to the city seeking medical advice—provided certain physicians are consulted.

THE DOCTOR AHEAD.—"If I were so unlucky," said an officer, "as to have a stupid son, I would certainly make him a doctor." "Well," said a doctor, who was in the company, "you think differently, sir, from your father."—*Medical Record.*

BROMIDIA.—Maurice Hache, M. D., 8 Rue de Tournon, Paris, May 18th, 1886, says: I have tried bromidia in two cases, one patient suffering from a slight febrile affection, the other a victim of acute insomnia; in the latter case various preparations of opium had proved useless and the administration of chloral was followed by lassitude and congestion in the head. Bromidia produced sound sleep in both of these cases, unaccompanied by any unpleasantness on awaking. In my opinion this preparation is destined to render good service, and I intend prescribing it whenever the opportunity presents itself.

ARSENIC IN SKIN DISEASES.—The editor of the *Journal of Cutaneous and Venereal Diseases* is desirous of ascertaining to what extent arsenic is used by American physicians in the treatment of skin diseases, and also the results of their experience as to its therapeutical value. Information upon the following points is requested of every physician who reads this: Are you in the habit of employing arsenic, *generally*, in the treatment of skin diseases? In what diseases of the skin have you found arsenic of superior value to other remedies? What ill effects, if any, have you observed from its use? What preparation of the drug do you prefer, and in what doses do you employ it? Address editor of *Journal of Cutaneous and Venereal Diseases*, 66 West 40th street, New York.

THERE are some doctors who are so closely watched by newspaper reporters that they cannot do any practice without its being found out and published.

A CORRECTION.—In THE JOURNAL for July, on page 290, first line, living should be *lining;* same page, fifth and sixth lines from bottom, inflammation should be *inflation.*

A BAD BOY.—A reporter of the *Evening Journal*, of this city, tells a good story on a stutterer. He says a man who stuttered badly accosted a small boy on Pryor street not long since in the following style: "C-ca-can y-y-you t-tell m-me wh-when th-the next t-t-train g-goes n-n-north, s-s-sonny?" The small boy responded, "12:40." The man then inquired: "H-h-have I g-g-got t-t-time to c-c-catch it?" The wicked boy replied with a grin: "Not unless you kin walk faster'n you kin talk."

MALTINE WITH CASCARA SAGRADA.—The Maltine Manufacturing Company, of 182 Fulton street, New York city, has just put before the profession this new combination as being superior to any other for the purpose for which it is intended. The value of cascara sagrada in all cases of obstinate and habitual constipation is conceded by nearly the entire profession. Combined with maltine, it becomes an agreeable and safe laxative, while the maltine invigorates and strengthens the whole system. Unlike ordinary purgatives, this compound causes no pain, nor does it leave the bowels in a weakened or enfeebled state, having, on the contrary, a tonic action. The improvement in health is noticed from the start. We quote from the remarks made by Dr. Reid, an eminent English practitioner, at a recent meeting of the British Medical Association, who said, regarding the use of cascara sagrada, in thirty-three cases of which he had taken careful notice, "the result was all that could be desired in twenty-seven cases of habitual constipation, several of them being complicated by various forms of dyspepsia, and in many instances the patients being persons of sedentary habits, more especially females." Also, the same authority states that cascara was found of great service in many cases of piles when other aperients had failed. Each fluid ounce of maltine contains one and a half drachms fl. ext. cascara sagrada. The medical profession is invited to send to the company for one pound of the above, which they will furnish without charge to those who will pay the express charge on the same.

SPONTANEOUS TRANSFORMATION OF MORPHINE INTO APOMORPHINE.—A solution of hydrochlorate of morphine for subcutaneous injection (3 per cent.) was ordered for a patient, and its injection was promptly followed by relief of the pain without any gastric symptoms whatever. Eleven months later, the patient made use of the same solution; but, this time, the injections gave rise immediately to violent and uncontrollable vomiting. The solution was given to a well-known analyst at Paris for examination, and he ascertained that apomorphine was present, thus accounting for the sickness. He recommended, in consequence, that solutions of the salts of morphine should never be kept longer than four weeks, and that freshly prepared solutions should not be mixed with the old.—*British Medical Journal, June 26.*

THE TREATMENT OF ULCERATIVE OTORRHŒA.—With a mirror on his forehead, and a good light thrown down a silver speculum therewith, the surgeon first cleanses the whole meatus thoroughly by means of little rolls of salicylic wool, wrapped round the end of a tapering probe. If this is thoroughly done, the granulations will be well seen in every part. Some of them may be so prominent as to form small polypi; others may be hardly at all raised. In either case, they must be scraped away freely with a sharp-edged curette and removed. The whole fundus of the ear is now cleansed and dried with small rolls of salicylic wool, as before; and, when quite dry, is touched freely with a roll of wool dipped in strong tincture of perchloride of iron. This fluid should be conveyed only to the ulcerating surface, and should be limited in amount. When it has remained in contact with the diseased area for a few seconds, it is dried off, and then a small quantity of iodoform, in fine powder, is blown over the part operated on. If this treatment be very carefully carried out in detail, ears, which have been discharging for months or years, may often be brought to heal in a few weeks. Everything depends on producing an aseptic, instead of a septic condition in the ears.—*Arthur E. Barker, in British Medical Journal.*

A LUXATED JAW REDUCED AFTER THREE MONTHS' STANDING.—Dr. Will B. Powell, Natchitoches, La., under date of May 21, thus writes: On May 13, 1878, a colored woman, aged 20 years, called on me for relief, stating her jaw (left side) was dislocated three months before during labor. She stated that she had puerperal convulsions. I made a careful examination; found left jaw completely thrown beneath the glenoid fossa, no fracture of process, condyle or symphysis. I placed patient under chloroform and tried in the usual way to reduce the luxation. I kept up pressure with the aid of spools of various sizes for nearly two hours without success. It being late I concluded to tie in the jaw a small spool, give a one-fourth grain of morphine, and wait until morning. On the 14th my patient returned, and again I tried, for at least a half hour, with no better success than the evening before. I might have done wrong in trying the experiment, but I conceived an idea that by throwing the right side out of place I could then reduce both sides at once. I took an empty spool (No. 30 Coates' spool) and placed it as far back as the first molars. My assistant pressed firmly downward, while I gave the jaw a smart blow from below upward, which produced the desired result. I now had a double luxation to reduce, which I accomplished by the ordinary plan without any trouble. I bandaged the jaw and discharged the patient, who reported in two weeks after as being quite well.

THE SCEPTIC.—A correspondent in the *British Medical Journal*, July 10th, writes: The great opponent to all progress in therapeutics is the sceptic. He never loses an opportunity of stating that he does not believe in therapeutics, "if there be such a thing." He publishes a book on medicine, and ostentatiously leaves out all reference to treatment. He declares openly that he does not know of any disease or morbid condition which can be influenced in the slightest degree by drugs. He does not credit any statements as to the good effect of iron in anæmia, and scoffs at the idea of mercury being of the slightest practical value in the treatment of syphilis. He has a deeply-rooted antipathy to phosphorus and arsenic, and considers anyone who prescribes them either a knave or a fool. In lecturing on the uses of such a drug as digitalis, he tells his audience that the dose of the infusion is from a teaspoonful to a bucketful; "it is all the same." When called upon to treat a case of poisoning, he advocates "expectant treatment," and is decidedly adverse to antidotes on principle. The sceptic is not, as a rule, well read; he is apt to think that every drug with which he is not familiar—and they are many—must needs be "an American quack remedy." The term "pharmacology" is not familiar to him, but he thinks it "has something to do with dispensing." By a curious coincidence, he is to be found on all hospital committees, and takes the greatest interest in the appointment of the lecturers on materia medica, preferring the candidate who knows least about it on the ground that he will have no preconceived ideas. The sceptic probably commenced life as a pathologist, but, after a variable period of time, has lost faith even in that. Having thrown his medical studies aside, he devotes his energy and attention to recondite works on philosophy. He gradually develops a disbelief in any future, and is doubtful about the past. At his necropsy, in which he possibly regrets being incapacitated from taking a part, a gumma is found in his brain, for which, years before, he had declined to be treated. Such are the men and the minds who so persistently oppose all progress in therapeutics. Unfortunately, their influence, pending the fatal termination, is often extremely prejudicial to any advance, and acts as a damper to the more enterprising and restless spirits, who, imbued with the enthusiasm of their calling, seek to obviate the present imperfections in our means of diagnosis and treatment, and have the courage of their opinions. The most disastrous effect is thus produced on the minds of the students, who are only too glad of an excuse to avoid the arid and difficult manipulations and study necessary to an intelligent comprehension of the facts at issue.

CHRONIC STARVATION.—Upon whatever other points they may differ, authorities on dietetics agree that nitrogen is the most essential of all foods, and that a certain amount should be taken regularly. Diminution of the quantity of food, whether from inability to procure it, or a disinclination for it, generally means decrease or absence of nitrogen. That this leads to dire results is a well established fact. Graily Hewitt, in an address on "Chronic Starvation and Delicate Females," before the British Medical Association, said: "For the last ten years or more I have carefully inquired into the history of patients suffering with uterine and ovarian disease, or some affection incidental to child-bed, and I have found a continuous insufficiency of food, especially the nitrogenous, to have existed almost universally, so that I have naturally come to regard this chronic starvation as an important factor in disease." Colden's Liquid Beef Tonic contains precisely the elements indicated by Dr. Hewitt as being so essential, combined with citrate of iron, cinchona, and simple aromatics, forming at once a palatable nutriment and reliable tonic, and its range of usefulness embraces all cases of debility of whatever origin. It has been in use fifteen years, and those who have used it most are most emphatic in its praise.—*Massachusetts Medical Journal*, Boston (Mass.), June, 1886.

PAINLESS REDUCTION OF SHOULDER DISLOCATIONS.—Let the patient lie down on his back on the floor or ground, with the dislocated arm outstretched at right angles to the trunk, and also on the floor. Having told the patient to lie quite still and make no effort, let the surgeon, placing the approximate heel in the axilla, make traction gently and steadily at right angles to the line of the trunk; and, as there may be no jerk or evident intimation of the return of the head of the hone to its place, let him ascertain its position, if necessary adducting the limb to make sure; if reduction has not taken place, let him renew and increase the force of traction, and repeat the examination until he has succeeded or failed, in which latter case nothing has been done to interfere with other methods. It is possible that, in many cases, the heel in the axilla may be unnecessary, but it will serve to steady the scapula, and affords a better counter-extending force than the weight of the patient's body, and thus leaves him free to lie still and make no effort as if to aid.—*Dr. MacLeod, in British Medical Journal.*

THE TREATMENT OF PUERPERAL CONVULSIONS WITH PILOCARPINE.—We have two or three times in previous issues alluded to the use of pilocarpine in puerperal eclampsia. Dr. Jas. Murphy, in the *Lancet* for May 29, 1886, publishes notes of three cases of convulsion occurring in the puerperal state, in all of which pilocarpine seemed to reduce the severity and frequency of the convulsions, and all of the cases recovered, a fact which by itself is sufficient to indicate that in all probability the recovery was partly, if not largely, to be attributed to the use of this remedy. The alkaloid was given in the dose of one-third of a grain injected hypodermically, as often as every six hours, or more frequently, if necessary.—*Therapeutic Gazette.*

DR. F. H. ORME, of this city, one of the most popular homœopathic physicians in the South, was recently elected President of the American Institute of Homœopathy.

PHYSICIANS in Georgia should use their influence against the election of any candidate to the next Legislature who voted against the bill "providing dissecting material for medical colleges," during the last session of the Legislature.

PEACOCK'S BROMIDES.—A. M. Chord, M. D., Logansport, Indiana, says: Peacock's bromides is a valuable remedy, and I can heartily recommend it to the profession where the use of such a preparation is indicated. It takes the place in our list of remedies that has long been needed. It is all that is claimed for it.

METEOROLOGICAL SUMMARY FOR JUNE, 1886, STATION, ATLANTA, GA.

| | |
|---|---|
| Mean Temperature, . . . . . . . . . . . . . . | 72.0 |
| Highest Temperature, June 12th, . . . . . . . . . | 90.0 |
| Lowest Temperature, June 5th, . . . . . . . . . . . | 60.0 |
| Monthly Range of Temperature, . . . . . . . . . . | 30.0 |
| Greatest Daily Range of Temperature, . . . . . . . | 20.0 |
| Least Daily Range of Temperature, . . . . . . . . . | 6.0 |
| Mean Daily Range of Temperature, . . . . . . . . . | 15.0 |
| Prevailing Direction of Wind, . . . . . . . . . . | S. E. |
| Total movement of wind, . . . . . . . . . . | 5,884 miles. |
| Highest Velocity of Wind and Direction, . . . | 32 Miles, S. |
| Total Precipitation. . . . . . . . . . . . | 8.68 inches. |
| Number of Days on which .01 inch or more of Rain fell . | 21.0 |
| Number of Foggy Days . . . . . . . . . . . . . . | .0 |
| " " Clear " . . . . . . . . . . . . . | 6.0 |
| " " Fair " . . . . . . . . . . . . . . | 12.0 |
| " " Cloudy " . . . . . . . . . . . . . . | 12.0 |
| Dates of Thunder-storms, . | 3, 6, 7, 8, 9, 10, 17, 24, 27, 28, 29 |

